# Changing indications for keratoplasty: monocentric analysis of the past two decades

**DOI:** 10.1007/s00417-024-06639-y

**Published:** 2024-09-14

**Authors:** Jan Vincent Hoffmann, Paola Kammrath Betancor, Philip Maier, Thabo Lapp, Sonja Heinzelmann, Daniel Böhringer, Stefan Lang, Thomas Reinhard

**Affiliations:** 1 Eye Center, Medical Center, University Freiburg, Killianstraße 5, 79106 Freiburg im Breisgau, Germany; 2 Department of Ophthalmology, St. Franziskus Hospital, Muenster, Germany; 3 Department of Ophthalmology, Brandenburg Medical School, University Hospital Brandenburg, Brandenburg an Der Havel, Germany

**Keywords:** Corneal graft registry, Keratoplasty, DMEK, Repeat keratoplasty, Corneal re-graft, Eye banking, Health care research

## Abstract

**Purpose:**

Over the past two decades, lamellar keratoplasty procedures, such as Descemet's Stripping Automated Endothelial Keratoplasty (DSAEK) and Descemet's Membrane Endothelial Keratoplasty (DMEK) as well as Deep Anterior Lamellar Keratoplasty (DALK), have become an integral part of clinical practice. With advances in contact lens fitting for keratoconus management and the implementation of UVA-Riboflavin Crosslinking (CXL), the landscape of keratoplasty indications is undergoing changes. Procedures and indications in a single tertiary care center over the past two decades are reviewed in this retrospective analysis. Methods: Retrospective analysis utilized anonymized electronic data from the LIONS cornea bank Baden-Württemberg, Eye Center Freiburg, spanning from beginning of 2004 to end of 2023. The primary focus was on surgical procedures and indications for keratoplasty.

**Results:**

The study encompassed a comprehensive analysis of 7130 corneal transplants. Penetrating keratoplasty (PK) exhibited a significant decline from 95% (*n* = 206, 2004) to 11% (*n* = 46, 2023), while DMEK increased from 48% (*n* = 157, 2012) to 82% (*n* = 347, 2023). Fuchs endothelial dystrophy (FED) emerged as the dominant indication, witnessing a significant increase from 24% (2004, *n* = 39) to 72% (2023, *n* = 288). Conversely, keratoconus (KC) showed a reciprocal change from 25% (2004, *n* = 40) to 4% (2023, *n* = 17). PK demonstrated a noteworthy indication shift in descending order from Bullous Keratopathy (BK), FED, and KC in 2004 to KC, graft failure, corneal scars, and ulcers in 2023. Repeat keratoplasty following DMEK remained rare, but a discernible upward trend was observed for PK.

**Conclusion:**

This retrospective analysis highlights significant changes in both keratoplasty indications and techniques over the past two decades. DMEK has emerged as a successful intervention for treating endothelial diseases, while PK retains its qualitative indispensability for a wide range of indications. Modern corneal banks are urged to maintain a robust supply of grafts for all surgical techniques in anticipation of potential increased demand in the future, particularly for repeat keratoplasties.

**Key messages:**

***What is known***

• Over the past two decades, lamellar keratoplasty procedures such as DSAEK and DMEK have increasingly replaced penetrating keratoplasty (PK) in clinical practice due to their improved outcomes and reduced complications for certain indications.

***New Findings***

• Our study reveals a significant shift in keratoplasty indications, with Fuchs endothelial dystrophy (FED) emerging as the predominant indication, increasing from 24% in 2004 to 72% in 2023, while keratoconus (KC) declined from 25 to 4% during the same period.

• Penetrating keratoplasty (PK) has shown a marked decline in use, dropping from 95% of keratoplasties in 2004 to 11% in 2023, whereas DMEK has grown to represent 82% of procedures in 2023.

• Despite the rise of DMEK, PK remains vital for a broad spectrum of indications, highlighting the necessity for corneal banks to maintain a versatile supply of grafts to meet diverse clinical needs, particularly in cases of repeat keratoplasties.

## Background

Keratoplasty, an essential surgical procedure to treat corneal disorders by replacing damaged or diseased corneal tissue, has a rich historical trajectory marked by the evolution of two primary techniques: penetrating as well as lamellar keratoplasty.

Penetrating keratoplasty finds its roots in the late nineteenth century, notably with Eduard Zirm's pioneering successful procedure in 1905 [1]. This landmark event served as a cornerstone, prompting continual refinements in surgical techniques, tissue preservation modalities, and post-operative care, continuously improving efficacy and success rates of penetrating keratoplasty [2].

The emergence of lamellar keratoplasty as an alternative approach to selectively replace affected corneal layers dates back to the 1940s [3]. Its rise has been aided by advances in microsurgical instrumentation and a deeper understanding of corneal anatomy. This technique has gained much attention and acceptance in the field with the aim of reducing the risk of graft rejection and better preserving the structural integrity of the host cornea [4].

The evolution of both penetrating as well as lamellar keratoplasty owes much to technological breakthroughs, including the integration of femtosecond laser technology for precise corneal dissection and the evolution of selective endothelial keratoplasty techniques [5–10].

Continuous research endeavors in keratoplasty strive to optimize surgical outcomes, mitigate complications, and broaden the spectrum of indications for corneal transplantation. These ongoing advances have positioned keratoplasty as a highly sophisticated and effective surgical modality for treating various corneal pathologies, profoundly improving visual acuity and overall quality of life for a global population [11–14].

As recently provided by Flockerzi E et. al. the performed keratoplasties in Germany were analyzed for 2011 to 2021. The data show the substantial increase in the importance of DMEK, which has now overtaken PK [15]. Thus, data show similar trends in German eye centers performing keratoplasties.

## Materials and methods

We analyzed the data of the LIONS cornea bank Baden-Württemberg, Eye Center Freiburg, with regards to indication of keratoplasty and surgery techniques. Approval by the Ethics Committee was given by the Albert-Ludwigs-University Freiburg (No. 23–1213-S1-retro). Anonymization of data was performed in accordance with the current data protection regulations prior to further investigations. A descriptive analysis of the collected data was then performed, taking into account the initial indication and distinguishing between different keratoplasty procedures for each year from 2004 to 2023. The respective data are displayed in line and bar charts as a function of the year. For evaluation of the proportion of therapeutic PK over all registered PK, they were defined as performed surgery within 7 days after listing on the waiting list. In addition, we obtained data on the rate of repeat keratoplasties depending on the surgical technique initially performed. Statistical analysis and data plotting was performed using R [16].

## Results

**Fig. 1:**
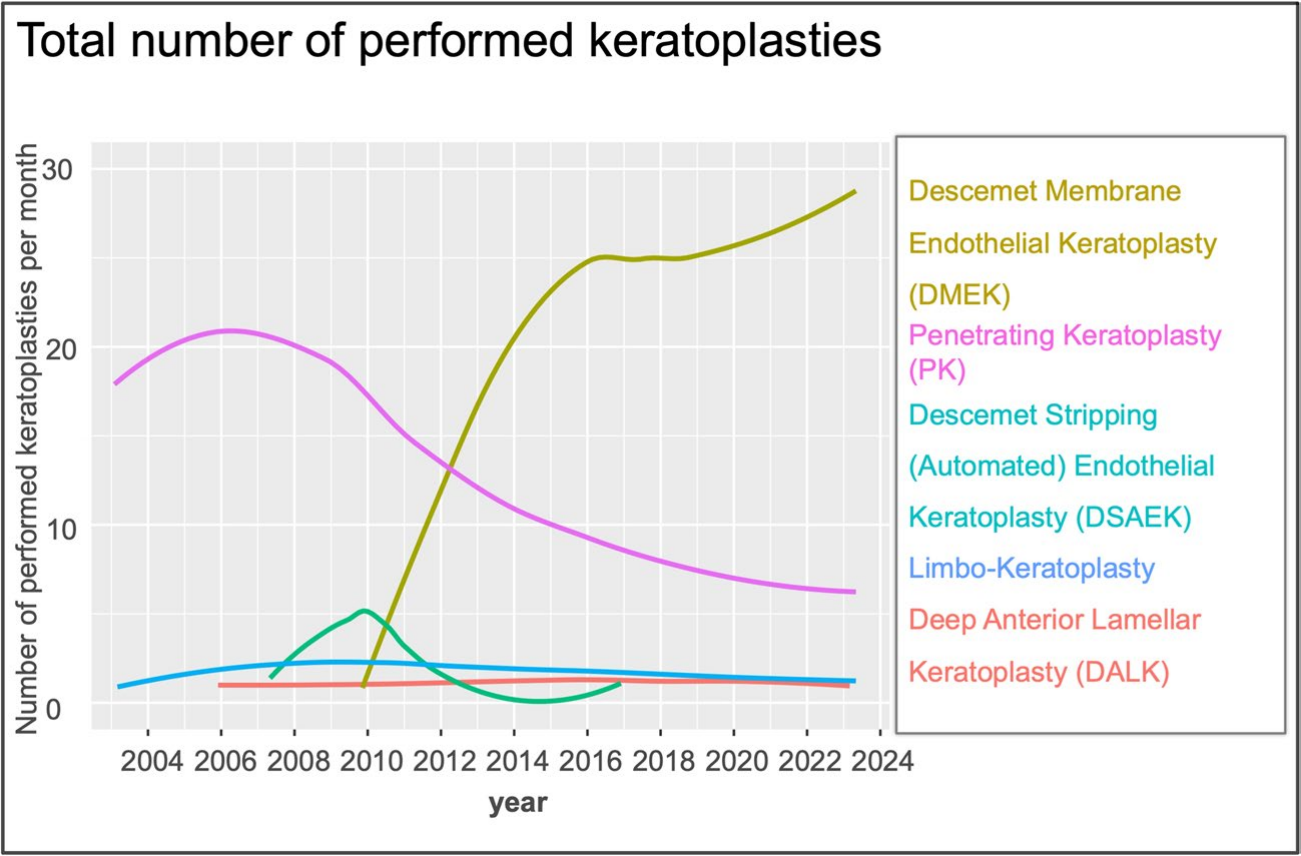
Absolute number of keratoplasties performed per month in the period from 2004 to 2023

We identified a total of 7130 keratoplasties for the period between 2004 and 2023. Overall, the number of corneal grafting procedures increased steadily, starting from 279 procedures in 2010 to 422 in 2023. The number of annual penetrating keratoplasties declined from 206 in 2004 to 46 in 2023. Considering the number of limbo-keratoplasties performed, it becomes apparent that there is no clear trend, as it has remained relatively stable at a low level. In the group of lamellar keratoplasties, DMEK has been the most frequently performed surgical technique since 2012. The number of DMEK performed rose steadily from 157 in 2012 to 347 in 2023 (Fig. 1). As the main representative of anterior lamellar keratoplasty, the number of DALKs undertaken has remained relatively consistent but at low levels. As far as grafts delivered to external centers are concerned, a steady decline has been observed over the last 20 years, although the decrease has been less pronounced in recent years. In the following charts, the grafts that were sent from our LIONS cornea bank Baden-Württemberg, Eye Center Freiburg, to external surgeons for transplantation are categorized as "external patients".

**Fig. 2:**
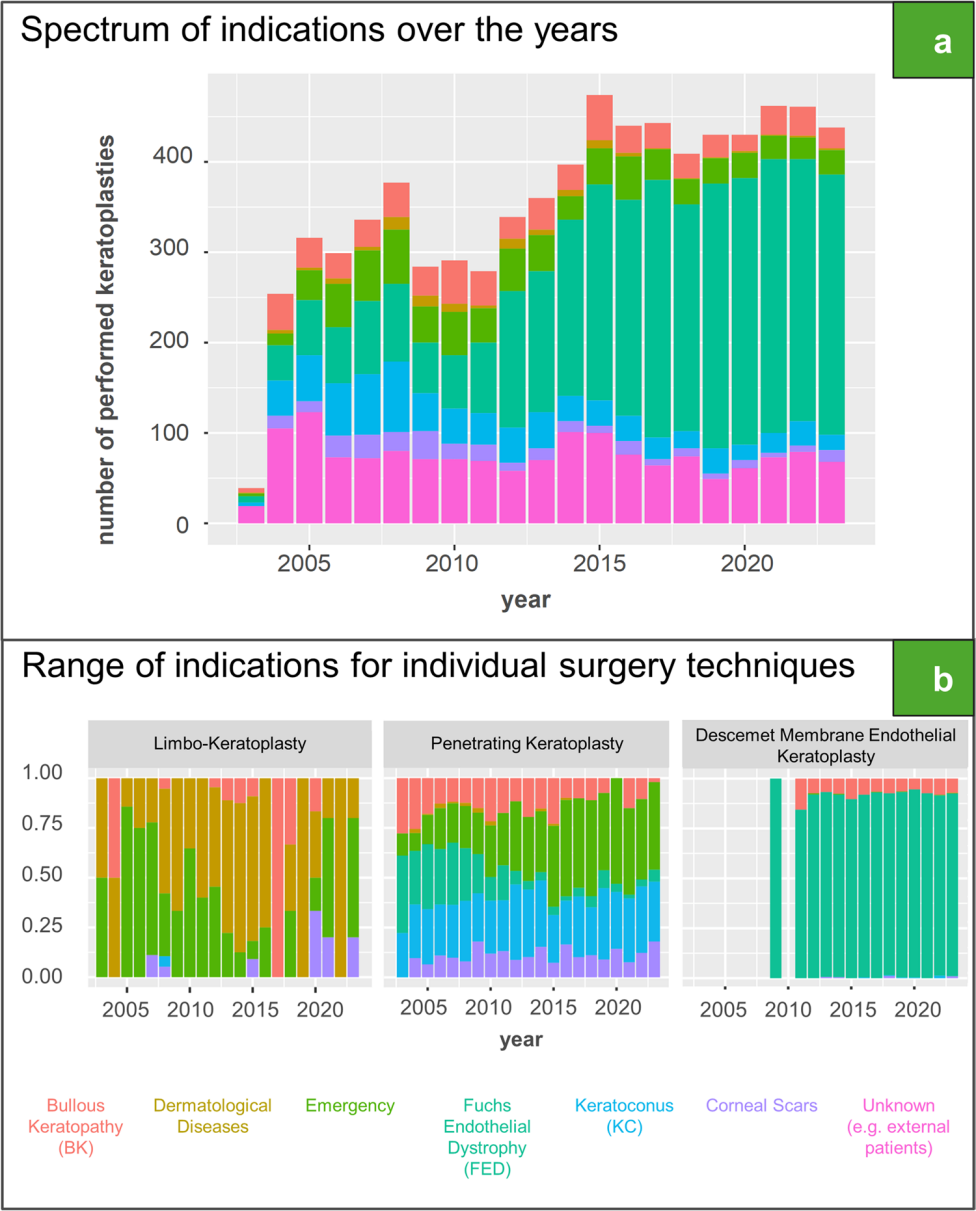
**a** Absolute proportion of the various indications of all keratoplasties performed from 2004 to 2023. *Dermatological Diseases* combine “blepharoconjunctivitis”, “pemphigoid” and “Stevens–Johnson syndrome”. *Emergency* consists of “tectonic”, “ulcer” and “trauma”. **b** Relative amount of the individual indications for limbo-keratoplasty, penetrating keratoplasty and Descemet Membrane Endothelial Keratoplasty

Quantitatively the two most important indications for corneal transplantation are FED with 3516 and KC with 733. With respect to these main indications, there was an increase from 39 (23%) in 2004 to 288 (68%) in 2023 for FED whereas a reciprocal development was determined for KC from 40 (15%) to 17 (4%). Further important indications were the BK as well as therapeutic indications such as perforated ulcers (Fig. 2A).

In 2004, the most relevant indications for PK were BK, FED and KC. There was a significant change in this spectrum over the years, mainly due to the fact the FED is now primarily treated by lamellar keratoplasty. Therefore, in 2023, the main indications were KC, graft failure, corneal scars and ulcers (Fig. 2B). Dermatological diseases, such as ocular pemphigoid, were treated with PK at a low level. In most cases, the only therapeutic option is still limbo-keratoplasty as shown in Fig. 2B or keratoprosthesis, e.g. Boston type 1 keratoprosthesis or osteo-odonto keratoprosthesis, performed in dedicated specialized eye centers [17–20].

**Fig. 3:**
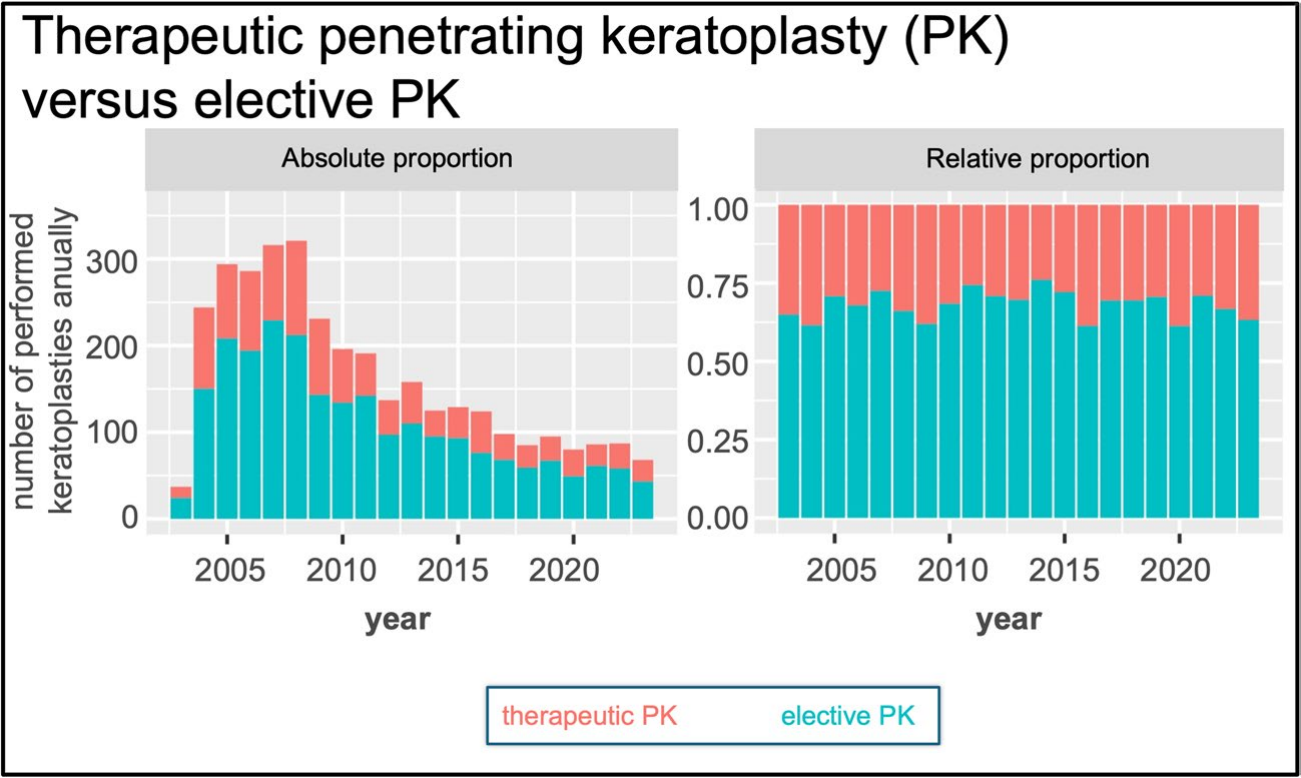
Absolute numbers (left) and relative proportions (right) of therapeutic penetrating keratoplasty (PK) among all PK procedures performed. A therapeutic PK was defined as a procedure in which the waiting time for the corneal graft did not exceed 7 days [21]

PK was also differentiated between elective PK and therapeutic PK. A comparison of the years 2004 to 2010 with 2011 to 2023 shows a decrease in the relative proportion of PK a chaud. Since 2011, there has been a relatively stable share of therapeutic PK (Fig. 3).

**Fig. 4:**
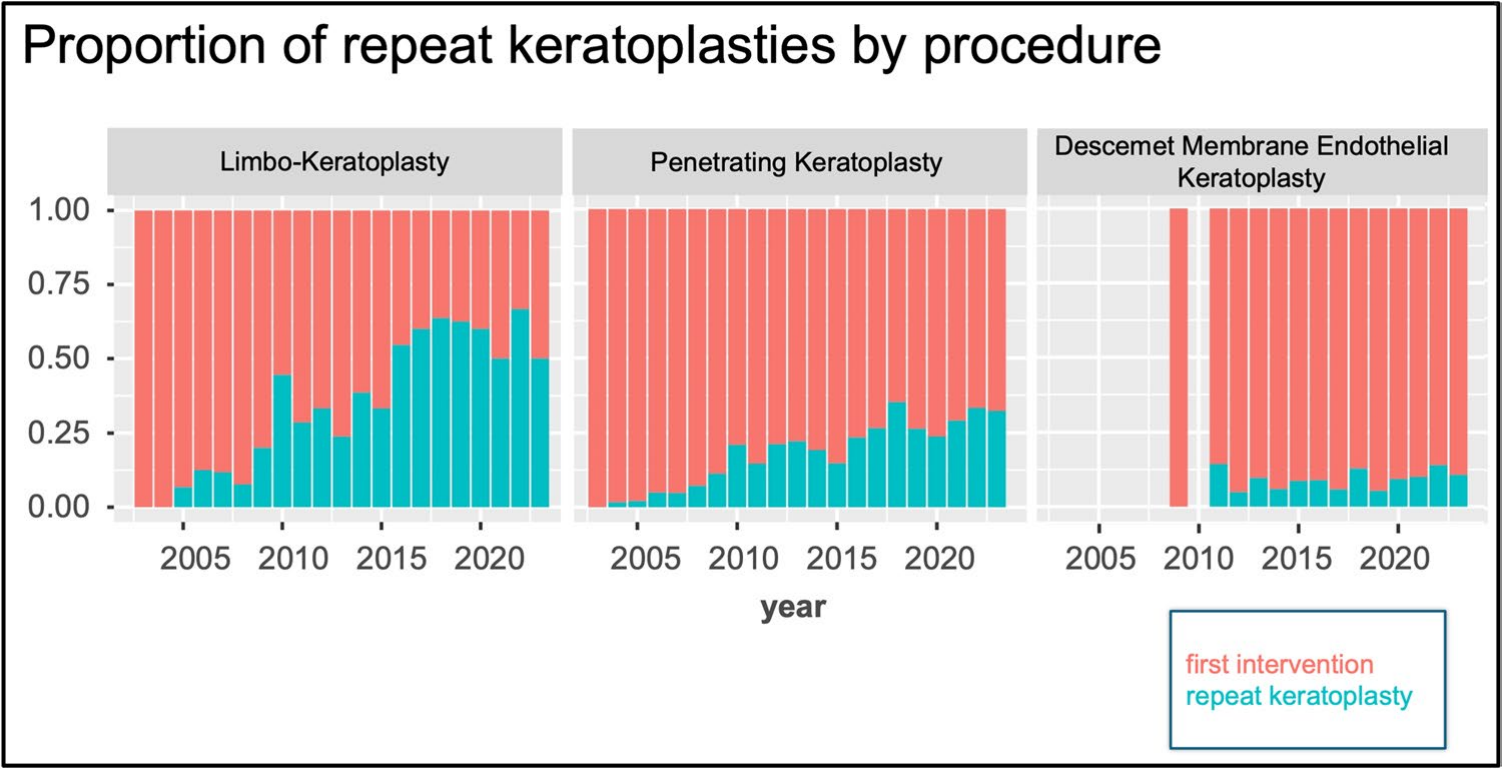
Relative proportion of initial procedures and repeat keratoplasties for limbo-keratoplasty, penetrating keratoplasty and Descemet Membrane Endothelial Keratoplasty in the period from 2004 to 2023

Furthermore, the proportion of repeat keratoplasties was analyzed depending on the surgical method initially performed. For DMEK repeated corneal transplantation was relatively stable from 2013 to 2023. However, the numbers of repeat PK are on the rise in the more recent years (Fig. 4).

**Fig. 5:**
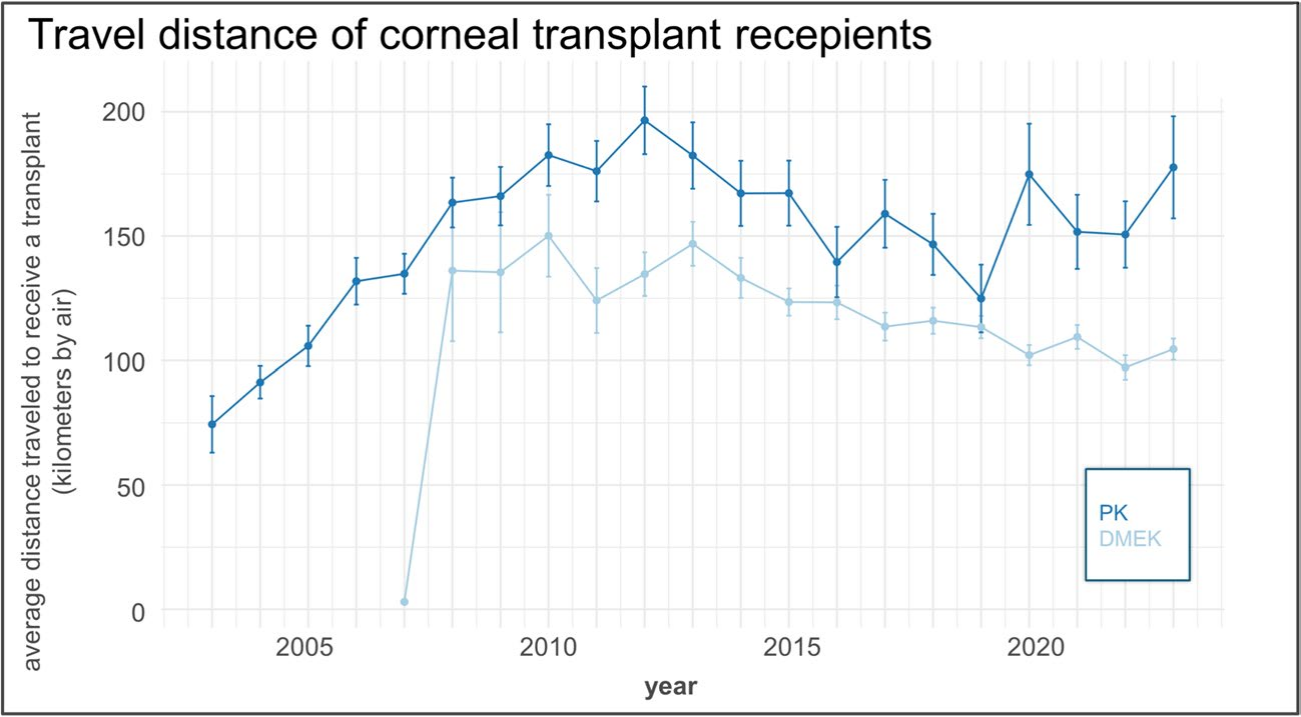
Air-line distance as a function of the recipients registered postal code for all keratoplasty techniques

Additionally, the air-line distance as a function of postal code of the recipient for all performed keratoplasties showed a significant increase from 2004 to 2010 with a corresponding peak of about 175 km. In the following years a decline to a steady state ranging between 100 to 125 km could be shown. Looking at the travel distance differentiated for PK, Limbo-KP and DMEK similar trends can be observed (Fig. 5).

## Discussion

This study analyses the change in indications for corneal transplantation over a period of twenty years and shows a significant change in the spectrum of indications, which is due, among other things, to a rapid change in surgical techniques. The decline in penetrating keratoplasties is matching the overall global trend [22–24]. The increase in the relative and absolute importance of DMEK is also in line with previously published data [25–27]. Looking at the change in the spectrum of indications leading to surgery, it is surprising that there is a reciprocal development with regard to KC. However, KC is expected to remain the main indication for penetrating keratoplasty. Moreover, PK will remain the gold-standard for therapeutic indications such as corneal ulcers. In other centers similar developments were described [28]. A recent retrospective multicenter analysis clearly shows that eye centers in Germany are now concentrating mainly on posterior lamellar keratoplasty and are primarily performing DMEK as its main representative. In North America and Australia, too, posterior lamellar keratoplasty has been performed predominantly in recent years. It is noticeable, however, that DS(A)EK is the most important method in these regions in the last years with a rising trend for DMEK. This trend was again confirmed in the report of the Eye Bank Association of America for 2023, with more DMEK than DSAEK being performed in the USA in that year for the first time. DALK, as the main representative of anterior lamellar keratoplasty, has been shown to have low but stable numbers over the years, suggesting that DALK retains its relevance for a small range of indications such as moderate keratoconus or anterior corneal scars without Descemet's membrane involvement. This trend is consistent with previously published data [15]. One common feature in all these regions, though, is that PKP is the second most common technology [15, 29–31]. Especially corneal ulcers can hardly be compensated by lamellar surgery techniques. With FED being the most important indication quantitatively, the increase of performed DMEK is not yet at its peak, and our data suggest that there will be an ongoing increase in the absolute number of DMEK performed in the coming years. Besides keratoprosthesis the only therapeutic option for existing bilateral limbal stem cell insufficiency, especially in chronic diseases such as ocular pemphigoid, is limbo-keratoplasty. Accordingly, the numbers have remained largely stable over the years, as there is still no established easy alternative, and the long-term results indicate a low graft rejection rate [32].

Comparing the results to other global regions it is striking, that in Northern America the relevance of bullous keratopathy leading to PK is still quantitatively the most important indication. We attribute this to the high quality standard and center-based cataract surgeons as well as the decline of anterior chamber intraocular lenses at our eye center, while in other regions, including the United States, this type of intraocular lens is still prevalent [33].

In the last decade, there was a relative increase of performed penetrating keratoplasties due to KC. This can best be explained by the fact, that the relative proportion of patients with FED leading to PK declined as DMEK is now the gold standard in this case [34].

It is worth noting that the number of PK leading to repeat keratoplasties has increased in recent years, while the proportion of repeat keratoplasties after initially performed DMEK has remained relatively stable. With regard to the shift of patients with FED, who are now usually treated initially with DMEK, it should be borne in mind that the proportion of complicated PKs is increasing and hence the relative number of repeat keratoplasties is rising accordingly.

While the total number of PK performed has decreased over the last two decades, the total number of therapeutic PK has also decreased accordingly. It has also been shown that the relative share of corneal grafts also experienced a negative trend during this period. Nevertheless, it must be emphasized that the proportion has remained relatively stable over the last 10 years. This clearly underlines how important it is for a corneal bank to keep a large variety of corneal buttons in stock, especially for therapeutic indications for performing a PK, as alternative procedures have not yet been established. It is important to note that as certain surgical methods decline in use, it is crucial to maintain the necessary training and surgical expertise to ensure the availability of these techniques when needed.

It must be taken into account that the monocentric design of this study only reveals the trends at LIONS BW cornea bank, Eye Center Freiburg, Nevertheless, similar trends as described above have been identified in other eye centers. It must also be considered that this is only a descriptive retrospective analysis. It is also important to bear in mind that no statement can be made about the indication and surgical technique etc. for transplants sent to external surgeons.

Considering the air-line distance using the postal codes of each patient in the years 2004 to 2010, the distance was constantly rising with a peak of approx. 175 km linear distance. From then on, travel distance slightly declined on a relatively high level of about 100 to 125 km. On the one hand, the data underlines the importance of the eye center for a large area, especially when new techniques such as DMEK are being established.

## Conclusion

In conclusion, there has been a significant increase in the absolute number of keratoplasties performed over the last two decades. This is partly due to the expansion of surgery indication, patient population, particularly with the introduction of DMEK for the treatment of FED, and partly due to the increased distance traveled by patients. The success of DMEK has also led to a significant change in the range of indications for which keratoplasty is performed, with endothelial keratopathies being the most common. However, it should not be forgotten that PK and DALK play an important role in the treatment of therapeutic indications as well as in the surgical management of KC when contact lens fitting or CXL is not successful.

## Abbreviations

BK: Bullous keratopathy
CXL: UVA-Riboflavin Crosslinking
DMEK: Descemet Membrane Endothelial Keratoplasty
DSAEK: Descemet's Stripping Automated Endothelial Keratoplasty
FED: Fuchs Endothelial Dystrophy
KC: Keratoconus
PK: Penetrating Keratoplasty
